# Stimmbezogene Lebensqualität und Emotionalität bei funktionellen Dysphonien in der Corona-Pandemie

**DOI:** 10.1007/s00106-025-01693-2

**Published:** 2025-12-09

**Authors:** Sven Schwenke de Wall, Frank Rosanowski, Ulrich Hoppe

**Affiliations:** 1 Audiologische Abteilung, Hals‑, Nasen‑, Ohrenklinik, Kopf- und Halschirurgie, Universitätsklinikum Erlangen und Friedrich-Alexander-Universität Erlangen-Nürnberg (FAU), Waldstraße 1, 91054 Erlangen, Deutschland; 2 Praxis für Phoniatrie, Pädaudiologie und HNO-Heilkunde, Kaiserstr. 35, 90403 Nürnberg, Deutschland; 3 Audiologische Abteilung, Hals‑, Nasen‑, Ohrenklinik, Kopf- und Halschirurgie, Universitätsklinikum Erlangen, Waldstraße 1, 91054 Erlangen, Deutschland

**Keywords:** COVID-19, Pandemien, Depressivität, Angst, Stress, COVID-19, Pandemics, Depression, Anxiety, Stress

## Abstract

**Hintergrund:**

Die Corona-Pandemie führte zu körperlichen Erkrankungen, seelischen Störungen, sozialen Problemen und noch nicht ausgeräumten gesellschaftlichen Konflikten. Dem Entzündungsgeschehen immanent waren organische Erkrankungen im HNO-Bereich und daraus folgten beeinträchtigende Funktionsstörungen, so Erkrankungen der Stimme als Folge morphologischer laryngealer Veränderungen. In der vorliegenden Arbeit ging es um das selbstempfundene Leiden an funktionellen Dysphonien in der Corona-Pandemie bei Patienten ohne auslösende COVID-Erkrankung unter der Annahme, dass die Situation in der Pandemie das Inanspruchnahmeverhalten der davon Betroffenen sowie ihre subjektive Bewertung gegenüber vor und nach der Pandemie veränderten.

**Material und Methoden:**

Grundlage der Studie waren die in der phoniatrisch-pädaudiologischen Facharztpraxis des Zweitautors in den Quartalen II/2019 bis einschließlich II/2023 erhobenen klinischen Routinedaten von insgesamt 972 erwachsenen Patienten, die sich erstmals wegen einer funktionellen Dysphonie vorgestellt hatten. Erhebungsinstrumente waren der Fragebogen zur stimmbezogenen Lebensqualität (Voice Related Quality of Life, V‑RQoL) sowie der DASS-Fragebogen (Depression Anxiety Stress Scales, Depressivitäts-Angst-Stress-Skalen) mit den Domänen Depressivität, Angst und Stress.

**Ergebnisse:**

Im Quartal vor dem ersten Lockdown war die Patientenzahl gegenüber dem Mittel aller anderen Quartale erhöht, während des Quartals des ersten Lockdowns erniedrigt. Im gesamten übrigen Beobachtungszeitraum schwankte die Patientenzahl in nichtsignifikantem Ausmaß. In allen gemessenen Domänen wurden keine signifikanten Unterschiede gegenüber der Zeit vor und nach der Pandemie festgestellt.

**Schlussfolgerung:**

Ein durch die Bedingungen der Pandemiezeit verändertes subjektives Leiden an funktionellen Dysphonien wurde weder störungsspezifisch im Hinblick auf die Stimmfunktion noch störungsübergreifend zur Emotionalität festgestellt.

## Abstract (en)

**Background:**

The COVID-19 pandemic led to physical illness, mental health disorders, social problems, and ongoing societal conflicts that have not yet been entirely resolved. Among the inflammatory processes associated with the virus, organic otorhinolaryngologic diseases occurred, resulting in functional impairments such as voice disorders caused by laryngeal affections. This study focused on the subjective suffering caused by functional dysphonia during the pandemic in patients who had not been affected by COVID-19. The underlying assumption was that the overall pandemic situation would alter both the healthcare-seeking behavior of affected individuals as well as their subjective assessments compared to the periods before and after the pandemic.

**Materials and methods:**

The study was based on clinical routine data collected from the phoniatric and pedaudiologic specialist practice of the second author covering the second quarter of 2019 through to the second quarter of 2023. A total of 972 adult patients who presented for the first time with functional dysphonia were included. Assessment tools were the Voice-Related Quality of Life (VRQoL) questionnaire and the Depression Anxiety Stress Scales (DASS) that measure depression, anxiety, and stress.

**Results:**

In the quarter preceding the first lockdown, the number of patients was higher than the average of all other quarters, while it was lower during the quarter of the first lockdown. For the remainder of the observation period, patient numbers fluctuated without statistical significance. Across all measured domains, no significant differences were observed when comparing the periods before and after the pandemic.

**Conclusion:**

No pandemic-related changes were found in the subjective experience of suffering from functional dysphonia, neither in terms of voice-specific dysfunction nor in emotional domains.

## Ätiologie und Inzidenz

Die Corona-Pandemie führte zu einer Fülle z. T. höchst beeinträchtigender körperlicher Erkrankungen, z. T. mit Todesfolge, zu psychischen Störungen und sozialen Problemen in allen Altersgruppen, außerdem zu gesellschaftlichen Konflikten über den Umgang mit der Pandemie, z. B. zur Frage des Lockdowns und zum Impfverhalten, deren politische Aufarbeitung auch im Hinblick auf das sinnvolle Vorgehen in möglichen zukünftigen Krisen dieser Art nicht abgeschlossen ist.

Während der Pandemie entstanden Erkrankungen des Kehlkopfs und Funktionsstörungen der Stimme und auch der (Phonations‑)Atmung bei an COVID erkrankten Patienten, z. B. durch die laryngeale Beteiligung am entzündlichen Geschehen [12, 28], möglicherweise bevorzugt als „Omicron-Laryngitis“ [10], durch morphologische Veränderungen, besonders in Folge einer Langzeitintubation und Beatmung in Bauchlage [4, 24], nach erfolgter Tracheotomie und/oder durch Interarytänoidfibrose und (sub)glottische Stenosen [12, 24]. Aber auch Motilitätsstörungen der Stimmlippen ohne erkennbare Ursache wurden beschrieben [12, 15]. Bei einer infektionsbedingten Lungenfunktionsstörung konnte die Stimme durch eine veränderte Phonationsatmung beeinträchtigt sein [13]. Nach einer COVID-Infektion wurden bei 70,2 % der Betroffenen anhaltende Stimmstörungen auch bei nicht zuvor hospitalisierten und nicht tracheotomierten Patienten festgestellt; nur bei 11,8 % davon konnte die Stimme konservativ therapeutisch geheilt, bei 64,7 % gebessert werden; bei 23,5 % konnte keine relevante Stimmkonsolidierung erreicht werden [28].

Auch bei nicht an COVID erkrankten Personen traten in der Pandemie vermehrt Stimmfunktionsstörungen und andere Vokaltraktbeschwerden auf: Sie betrafen z. B. Lehrer im Distanzunterricht [3] sowie das Personal im Gesundheitswesen und in Pflegeeinrichtungen, das permanent Atemschutzmasken tragen musste [8, 26].

Das Leiden an einer Stimmstörung, operationalisiert als Voice Handicap Index oder als verminderte stimmbezogene Lebensqualität, gemessen anhand des Fragebogens Voice-Related Quality of Life, V‑RQoL [2, 7, 13], scheint bei Patienten mit morphologischen Ursachen nach einer COVID-Erkrankung besonders ausgeprägt zu sein, am ehesten wegen der in diesen Fällen stärker beeinträchtigten Stimmfunktion [24]. Die Schwere einer durchgemachten COVID-Erkrankung sowie ein höheres Lebensalter bedingen ein ausgeprägteres Leidensgefühl auch im Hinblick auf die Stimmfunktion; ein anderer möglicher ursächlicher Faktor für eine größer empfundene Beeinträchtigung ist das notwendige Tragen von Atemschutzmasken, besonders bei rauchenden Frauen in stimmintensiven Berufen [18]. Eine spezifisch „psychosomatische“ Dysphonie bildet sich in der Zunahme der Patientenzahl mit einer „inducible laryngeal obstruction“ (frühere Bezeichnung: „vocal cord dysfunction“) während der Pandemie ab mit der Verdoppelung ihrer Inzidenz auf 10,3 % in einem Kollektiv von insgesamt 1243 Personen einer Spezialambulanz [1].

Das stimmbezogene Leiden von Patienten mit funktionellen Dysphonien während der Pandemie, aber ohne eigene COVID-Vorerkrankung, wurde bisher nicht gezielt untersucht. Spezifische emotionale Aspekte hingegen, nämlich Ängstlichkeit und Depressivität in der Pandemiezeit, wurden an einer Kohorte älterer Menschen im Zusammenhang mit deren Kommunikationsverhalten beschrieben: Dabei empfanden kommunikativ aktivere Personen mit einer gleichzeitigen Angstbelastung ein größeres Stimmhandicap, das galt vermehrt bei den älteren Studienteilnehmern [29].

Dem Anliegen einer psychosomatischen Grundeinstellung folgend werden auch bei Patienten mit Stimmerkrankungen neben dem Organ- und Funktionsbefund psychosoziale Aspekte gleichwertig berücksichtigt, auch weil dies die Zufriedenheit der betroffenen Patienten steigert [2, 20, 22, 23]. In der vorliegenden Studie geht es um diese „subjektive Seite“ von Stimmerkrankungen während der Corona-Pandemie:

Konkret wurde an nichtselektionierten Patienten einer phoniatrisch-pädaudiologischen Facharztpraxis sowohl Veränderungen der Patientenzahl, der Geschlechts- und Altersstruktur sowie – mit dem psychosomatischen Fokus – mögliche Veränderungen der stimmbezogenen Lebensqualität und emotionaler Aspekte (Depressivität, Ängstlichkeit, Stress) bei Patienten mit nicht organisch bedingten, sog. funktionellen Dysphonien in der Pandemie untersucht und mit Daten aus der Zeit vor und nach der Pandemie verglichen. Aus den Ergebnissen wurden Rückschlüsse auf ein verändertes Inanspruchnahmeverhalten sowie auf ein pandemiebedingt verändertes Leiden in der Pandemiezeit hergeleitet.

## Material und Methoden

Grundlage der Studie waren die in der phoniatrisch-pädaudiologischen Facharztpraxis des Zweitautors in den Quartalen II/2019 bis einschließlich II/2023 erhobenen klinischen Routinedaten von insgesamt 972 erwachsenen Patienten, die sich erstmals wegen einer funktionellen Dysphonie vorgestellt hatten. Patienten mit einer anamnestisch durchgemachten COVID-Erkrankung wurden nicht inkludiert. Insgesamt handelte es sich um 652 Frauen, entsprechend 67,1 % des Gesamtkollektivs, und 320 Männer, entsprechend 32,9 %. Die Geschlechtsverteilung war in den einzelnen Quartalen nahezu unverändert (Tab. 1).

**Tab. 1:** Alters- und Geschlechtsverteilung von Patienten mit funktionellen Dysphonien im Untersuchungszeitraum 2. Quartal 2019 bis 2. Quartal 2023.

	II 2019	I 2020	II 2020	III 2020	IV 2020	I 2021	II 2021	III 2021	IV 2021	II 2022	II 2023
Gesamt	83	114	65	88	77	88	99	90	97	96	79
Männer	23 (28 %)	38 (33 %)	21 (22 %)	31 (35 %)	30 (39 %)	27 (31 %)	35 (38 %)	29 (32 %)	28 (29 %)	28 (29 %)	30 (39 %)
Frauen	60 (72 %)	76 (67 %)	44 (78 %)	57 (65 %)	47 (61 %)	61 (69 %)	61 (62 %)	61 (68 %)	69 (71 %)	68 (71 %)	48 (61 %)
MW Alter (Jahre)	53,6	51,8	50,1	51,1	47,4	50,0	51,7	51,5	51,5	49,5	53,0
STD Alter (Jahre)	14,9	16,9	16,4	17,2	15,7	17,2	18,6	16,4	18,1	15,7	15,2

*MW* Mittelwert, *STD* Standardabweichung

Das mittlere Lebensalter war im Gesamtkollektiv 51,01 ± 16,60, der Bereich war 18 bis 89 Jahre. Bei den Frauen betrug es 51,06 ± 16,67 mit einem Bereich von 18 bis 89 Jahren. Bei den Männern betrug es 51,15 ± 16,69 mit einem Bereich von 18 bis 87 Jahren. Das Alter der Frauen und der Männer war im Gesamtzeitraum und in den einzelnen Quartalen statistisch nicht signifikant unterschiedlich (*T*-Test: *p* = 0,94). Die Altersverteilung in den einzelnen Quartalen war statistisch nicht signifikant unterschiedlich (Kruskal-Wallis-Test: H(10) = 9,11; *p* = 0,52).

In den einzelnen Quartalen wurden jeweils zwischen 65 und 114 Patienten untersucht. Die Patientenzahl im Quartal I/2020 war statistisch signifikant erhöht, im Quartal II/2020 statistisch signifikant reduziert gegenüber dem Mittelwert der jeweils anderen Quartale (*T*-Test: *p* < 0,0001 bzw. *p* < 0,0001).

Zur Erhebung der stimmbezogenen Lebensqualität wurde der Fragebogen Voice-Related Quality of Life (V-RQoL) in seiner deutschsprachigen Version eingesetzt [7]. Zunächst sollen die Patienten den empfundenen Zustand ihrer Stimme auf einer vierstufigen Skala einschätzen. Die Antworten auf ihr Erleben der Beeinträchtigung in spezifischen Kommunikationssituationen wird in einen Prozentwert überführt; hohe Prozentwerte stehen für eine große stimmbezogene Lebensqualität [27]. Die Fragen und die Bearbeitungshinweise für die Patienten sind in Tab. 2 aufgelistet.

**Tab. 2:** Fragebogen zur stimmbezogenen Lebensqualität (Voice Related Quality of Life, V‑RQoL) [7, 27]. Vorangestellt ist folgende Anrede: „Liebe Patienten, der vorliegende Fragebogen soll uns dazu dienen, mehr über die Auswirkungen Ihrer Stimmprobleme auf Ihr tägliches Leben zu erfahren. Diese Information ist zum einen notwendig, um Ihr Stimmproblem richtig einschätzen zu können. Außerdem benötigen wir im Falle einer Nachfrage des Kostenträgers vernünftige Argumente, warum Ihr Stimmproblem überhaupt untersucht und ggf. auch behandelt werden muss. Der Fragebogen enthält eine Liste von möglichen Problemen, die mit Ihrer Stimme in Zusammenhang stehen könnten. Bitte beantworten Sie alle Fragen unter dem Gesichtspunkt, wie die Verfassung Ihrer Stimme während der letzten zwei Wochen war. Bitte antworten Sie unbefangen; es gibt keine ‚richtigen‘ oder ‚falschen‘ Antworten. Bitte geben Sie für jede der Aussagen auf der Liste an, wie zutreffend das dort geschilderte Problem für Sie ist: Bewerten Sie also das Ausmaß jedes der genannten möglichen Probleme. Berücksichtigen Sie dabei sowohl die Schwere des Problems als auch die Häufigkeit seines Auftretens.“

*Bitte kreuzen Sie das Wort an, das den Zustand Ihrer Stimme Ihrer Meinung nach heute am besten beschreibt:*						
Normal ◯						
Leicht gestört ◯						
Mittelmäßig gestört ◯						
Schwer gestört ◯						
*Kreuzen Sie bitte jeweils die für Sie am ehesten zutreffende Antwort an:*						
Bitte benutzen Sie bei der Beantwortung der folgenden Fragen die folgende Skala:						
**1** = kein Problem, **2** = kaum ein Problem, **3** = schon ein Problem, **4** = ein großes Problem, **5** = ein Problem, wie es schlimmer nicht sein kann						
Wie ausgeprägt ist dieses Problem für Sie?						
1.	Meine Stimme erschwert es mir, laut zu sprechen oder mir in einer lauten Umgebung Gehör zu verschaffen.	1	2	3	4	5
2.	Beim Sprechen gerate ich außer Atem und muss oft nach Luft schnappen.	1	2	3	4	5
3.	Wenn ich zum Sprechen ansetze, weiß ich oft nicht, was ich an Lauten hervorbringen werde.	1	2	3	4	5
4.	Wegen meiner Stimme bin ich manchmal befangen oder frustriert.	1	2	3	4	5
5.	Manchmal fühle ich mich aufgrund meines Stimmproblems niedergeschlagen.	1	2	3	4	5
6.	Meine Stimme ist mir beim Telefonieren hinderlich.	1	2	3	4	5
7.	Meine Stimme behindert mich bei der Ausübung meines Berufs.	1	2	3	4	5
8.	Wegen meiner Stimme meide ich gesellige Aktivitäten.	1	2	3	4	5
9.	Um mich verständlich zu machen, muss ich mich wiederholen.	1	2	3	4	5
10.	Wegen meiner Stimme bin ich jetzt weniger kontaktfreudig als früher.	1	2	3	4	5

Die Emotionalität wurde mit dem DASS-Fragebogen (Depression Anxiety Stress Scales, Depressivitäts-Angst-Stress-Skalen) erhoben [16]. Die Auswertung des DASS-Fragebogens basiert auf den Rohwerten, hohe Punktwerte stehen für ein großes Belastungsgefühl in den drei Subskalen. Ein „Gesamtwert“ wird nicht gebildet. Der Fragebogen einschließlich der Zuordnung zu den drei Subskalen sowie die Bearbeitungshinweise für die Patienten sind in Tab. 3 aufgeführt.

**Tab. 3:** DASS-Fragebogen zu Depressivität, Angst und Stress nach Nilges und Essau [16].

*Bearbeitungshinweis: *Bitte lesen Sie jede Aussage und kreuzen Sie die Zahl 0, 1, 2 oder 3 an, die angeben soll, wie sehr die Aussage *während der letzten Woche *auf Sie zutraf. Es gibt keine richtigen oder falschen Antworten. Versuchen Sie, sich spontan für eine Antwort zu entscheiden.						
0 Traf *gar nicht *auf mich zu						
1 Traf *bis zu einem gewissen Grad *auf mich zu oder *manchmal*						
2 Traf *in beträchtlichem Maße *auf mich zu oder *ziemlich oft*						
3 Traf *sehr stark *auf mich zu oder *die meiste Zeit*						
1.	Ich fand es schwer, mich zu beruhigen.	0	1	2	3	*S*
2.	Ich spürte, dass mein Mund trocken war.	0	1	2	3	*A*
3.	Ich konnte überhaupt keine positiven Gefühle mehr erleben.	0	1	2	3	*D*
4.	Ich hatte Atemprobleme (z. B. übermäßig schnelles Atmen, Atemlosigkeit ohne körperliche Anstrengung).	0	1	2	3	*A*
5.	Es fiel mir schwer, mich dazu aufzuraffen, Dinge zu erledigen.	0	1	2	3	*D*
6.	Ich tendierte dazu, auf Situationen überzureagieren.	0	1	2	3	*S*
7.	Ich zitterte (z. B. an den Händen).	0	1	2	3	*A*
8.	Ich fand alles anstrengend.	0	1	2	3	*S*
9.	Ich machte mir Sorgen über Situationen, in denen ich in Panik geraten und mich lächerlich machen könnte.	0	1	2	3	*A*
10. Ich hatte das Gefühl, dass ich mich auf nichts mehr freuen konnte.		0	1	2	3	*D*
11. Ich bemerkte, dass ich mich schnell aufregte.		0	1	2	3	*S*
12. Ich fand es schwierig, mich zu entspannen.		0	1	2	3	*S*
13. Ich fühlte mich niedergeschlagen und traurig.		0	1	2	3	*D*
14. Ich reagierte ungehalten auf alles, was mich davon abhielt, meine momentane Tätigkeit fortzuführen.		0	1	2	3	*S*
15. Ich fühlte mich einer Panik nahe.		0	1	2	3	*A*
16. Ich war nicht in der Lage, mich für irgendetwas zu begeistern.		0	1	2	3	*D*
17. Ich fühlte mich als Person nicht viel wert.		0	1	2	3	*D*
18. Ich fand mich ziemlich empfindlich.		0	1	2	3	*S*
19. Ich habe meinen Herzschlag gespürt, ohne dass ich mich körperlich angestrengt hatte (z. B. Gefühl von Herzrasen oder Herzstolpern).		0	1	2	3	*A*
20. Ich fühlte mich grundlos ängstlich.		0	1	2	3	*A*
21. Ich empfand das Leben als sinnlos.		0	1	2	3	*D*

Die Dokumentation und Auswertung der retrospektiv erhobenen Daten erfolgte anonymisiert mittels Excel® (Microsoft, Redmond, WA, USA) und Matlab® R2019 (MathWorks, Natick MA, USA). Das Vorhaben wurde von der Ethik-Kommission der Medizinischen Fakultät der Friedrich-Alexander-Universität Erlangen-Nürnberg genehmigt (Geschäftszeichen 24-60-Br).

## Ergebnisse

### Fragebogen zur stimmbezogenen Lebensqualität (Voice-Related Quality of Life, V-RQoL)

Der selbst empfundene Zustand der Stimme wurde von insgesamt 199 Patienten (20,6 %) als normal, von 381 (39,4 %) als leicht gestört, von 306 (31,6 %) als mittelmäßig gestört und von 82 (8,4 %) als schwer gestört angegeben. Die Antworten von Frauen und Männern waren im Gesamtkollektiv statistisch nicht signifikant unterschiedlich (*T*-Test: *p* = 0,1215). In den einzelnen Quartalen waren die Antworten nicht statistisch signifikant unterschiedlich (Kruskal-Wallis-Test: H(10) = 16,8; *p* = 0,079), bei den Frauen war das *p* = 0,259; bei den Männern war das *p* = 0,081.

Im Gesamtkollektiv unterlag die Einschätzung des Zustands der Stimme einer Altersabhängigkeit: Je älter die Patienten waren, desto ausgeprägter empfanden sie das Ausmaß ihrer Stimmstörung (r = 0,11; *p* = 0,0063, Spearman-Rangkorrelationskoeffizient). Bei Frauen konnte keine Altersabhängigkeit nachgewiesen werden (r = 0,076; *p* = 0,053), hingegen bei Männern (r = 0,17; *p* = 0,002).

Für die einzelnen Quartale wurde die Altersabhängigkeit des selbst empfundenen Ausmaßes der Stimmstörung wegen der kleinen Subgruppen nicht analysiert.

Der Prozentwert der stimmbezogenen Lebensqualität betrug im Gesamtkollektiv bei Frauen 68,6 ± 22,5 % und bei Männern 73,4 ± 21,5 %; im Gesamtkollektiv war dieser Unterschied statistisch signifikant (*T*-Test: *p* = 0,0015; Rangsummentest *p* = 0,0012). Beim Vergleich der in den einzelnen Quartalen erhobenen Werte lagen zwischen Frauen und Männern keine statistisch signifikanten Unterschiede vor (Kruskal-Wallis-Test: H(10) = 14,81; *p* = 0,14).

Der Prozentwert der stimmbezogenen Lebensqualität war im Gesamtkollektiv statistisch nicht signifikant vom Lebensalter abhängig (r = 0,05; *p* = 0,133; Spearman-Rangkorrelationskoeffizient). Bei Frauen war er signifikant (r = 0,09; *p* = 0,02), bei den Männern nicht (r = −0,04; *p* = 0,50).

Für die einzelnen Quartale wurde die Altersabhängigkeit des Prozentwerts der stimmbezogenen Lebensqualität wegen der kleinen Subgruppen nicht analysiert.

### DASS-Fragebogen zu Depressivität, Angst und Stress

Auf der Subskala Depressivität D lag der Mittelwert bei Frauen im Gesamtkollektiv bei 3,6 ± 4,5 und bei Männern bei 3,3 ± 4,3. Auf der Subskala Angst A lag der Mittelwert bei Frauen bei 4,6 ± 4,3 und bei Männern bei 4,0 ± 3,8. Auf der Subskala Stress S lag der Mittelwert bei Frauen bei 5,4 ± 4,9 und bei Männern bei 4,9 ± 4,9. Die Unterschiede zwischen Frauen und Männern waren im Gesamtkollektiv für D und S nicht signifikant (*p* = 0,25 und *p* = 0,14), jedoch für A (*p* = 0,02; *T*-Test).

Eine Altersabhängigkeit des Antwortverhaltens lag bei allen Subskalen weder im Gesamtkollektiv noch bei getrennter Auswertung von Frauen und Männern vor (Spearman-Korrelation *p* > 0,14).

In den einzelnen Quartalen waren die Antworten für die Subskalen Depressivität und Angst nicht statistisch signifikant unterschiedlich (Kruskal-Wallis-Test: H(10) = 15,84; *p* = 0,104 bzw. H(10) = 14,66; *p* = 0,145). Für die Subskala Stress wurde ein unterschiedliches Antwortverhalten über die Quartale gefunden (Kruskal-Wallis-Test: H(19) = 21,22; *p* = 0,019). Die Mediane für die Subskala Stress lagen in den Quartalen zwischen 3 und 5. Eine Zuordnung zum Pandemieverlauf war dabei nicht erkennbar.

### Zusammenhang zwischen der stimmbezogenen Lebensqualität sowie Depressivität, Ängstlichkeit und Stress

Im Gesamtkollektiv war der Prozentwert der stimmbezogenen Lebensqualität mit dem Wert der Depressivität mit r = −0,41 und *p* < 10^−6^ statistisch signifikant korreliert, mit dem Wert der Ängstlichkeit mit r = −0,40 und *p* < 10^−6^ und mit dem Wert der Subskala Stress mit r = −0,41 und *p* < 10^−6^ (Spearman-Rangkorrelationskoeffizient). Dieser statistisch signifikante Zusammenhang galt für alle drei Subskalen auch in allen einzelnen Quartalen (*p* < 0,01). Beim Vergleich von Frauen und Männern fand sich in allen Quartalen kein Unterschied der Korrelationen (r zwischen −0,47 und −0,38, *p* < 10^−6^).

## Diskussion

Die Auswahl des Zeitraums für die Datenanalyse dieser Studie orientierte sich an den Wellen der Corona-Pandemie und folgt dem Vorgehen in anderen Studien [21]. Die meisten phoniatrisch-pädaudiologischen Einrichtungen arbeiten in der ambulanten Versorgung, niedergelassene Fachärzte machen ihren größten Anteil aus [32]. Auch vor dem Hintergrund der hinreichend groß erscheinenden Fallzahl werden die Repräsentativität der Studienergebnisse und ihre Übertragbarkeit auf ähnliche Einrichtungen und Praxen angenommen.

Die Patienten mit funktionellen Dysphonien stellen sich in der der berichtenden phoniatrisch-pädaudiologischen Facharztpraxis nahezu vollständig auf Überweisung eines HNO-Arztes vor, so auch während der Pandemie. Eine Fokussierung der Zuweiser auf organische Stimmstörungen und eine in den Hintergrund tretende Bedeutung funktioneller Dysphonien lässt sich aus den erhobenen Daten nicht herleiten.

Im Quartal I/2020, also unmittelbar vor dem ersten Lockdown in Deutschland (22.03.2020 bis 04.05.2020), war die Patientenzahl gegenüber dem Durchschnitt der anderen Quartale erhöht, möglicherweise wegen des Patientenwunsches nach einer fachärztlichen Klärung ihrer Beschwerden vor den bereits damals diskutierten bzw. zu erwartenden Einschränkungen der Mobilität. Im zweiten Quartal 2020, also während der Zeit des ersten Lockdowns, war die Patientenzahl erniedrigt, wohl als Ausdruck der Konformität mit den geltenden Einschränkungen und deren Begründung. In allen weiteren Quartalen während der Pandemie war die Patientenzahl gegenüber dem Durchschnitt unverändert. Offenbar hatten die Betroffenen einen genügend großen Leidensdruck, und/oder sie vertrauten auf ein pandemiekonformes Verhalten der Praxis.

Nach Literaturangaben wurden Larynxkarzinome während der Pandemie später diagnostiziert – mit dann ausgedehnteren Tumoren [6]; Prokein et al. hingegen fanden in einem deutschen Kollektiv keine Veränderung der Tumormeldezahlen und sogar auch eine Zunahme der kleineren Tumorstadien beim Vergleich mit präpandemischen Daten [19]. Bei anderen „typischen“ phoniatrisch-pädaudiologischen Patienten wie z. B. Kindern mit dem Verdacht auf das Vorliegen einer auditiven Verarbeitungs- und Wahrnehmungsstörung (AVWS) wurde in der berichtenden Praxis während des ersten Pandemiejahres und auch noch im Jahr danach eine geringere Patientenzahl vorstellig als zuvor. Veröffentlichungen zu potenziell akut lebensbedrohlichen Erkrankungen jenseits des Fachgebiets wie Herzinfarkt und Apoplex sind uneinheitlich im Hinblick auf in der Pandemie veränderten Patientenzahlen (Diskussion dazu bei [9]). Insgesamt lässt sich zur Inanspruchnahme des Versorgungssystems während der Pandemie kein durchgängiges Patientenverhalten und kein daraus erwachsender veränderter Outcome erkennen, auch wenn die Zunahme psychischer Störungen besonders im Kindes- und Jugendalter während und anhaltend nach der Pandemie einen deutlich gesteigerten Bedarf an veränderten Versorgungsstrukturen nahelegt [17, 21].

Ein „geschlechtsspezifisch“ anderes Inanspruchnahmeverhalten sowie eine andere Altersverteilung als außerhalb der Pandemie konnten in der vorliegenden Studie nicht beobachtet werden, sie waren identisch.

Der Fragebogen zur stimmbezogenen Lebensqualität (Voice-Related Quality of Life, V‑RQoL) ist international gebräuchlich und wird in seiner deutschen Version auch in der aktuellen Leitlinie als ein mögliches Diagnoseverfahren genannt, er ist dem sog. Voice-Handicap-Index (VHI) gleichwertig [2, 5, 7, 30, 31]. Beide bilden zuverlässig eine subjektive Veränderung bei und nach einer Stimmtherapie ab mit einer Korrelation zum Stimmstatus, auch bei Patienten mit Dysphonien als Teil einer Long- bzw. Post-COVID-Problematik [28]. Die Erhebung der emotionalen Kategorien Ängstlichkeit, Depressivität und Stress folgt dem psychosomatischen Ansatz, neben dem Organ- und Funktionsbezug psychosoziale Aspekte gleichwertig zu berücksichtigen [23]. Die Anwendung eines störungsspezifischen und eines „störungsübergreifenden“ Diagnostikums kann Maßnahmen der psychosomatischen Grundversorgung auch bei Patienten mit Dysphonien leiten und steigert zudem die Patientenzufriedenheit [20]. Beide hier eingesetzten Fragebögen sind frei verfügbar [7, 16].

Ein gegenüber der Zeit vor und nach der Pandemie verändertes Antwortverhalten im Fragebogen zur stimmbezogenen Lebensqualität wurde im Kollektiv dieser Studie nicht festgestellt. Ein individuelles Ursachengefüge mit einer initialen COVID-Infektion war ausgeschlossen und scheidet als möglicher konfundierender Faktor aus, da solche Patienten nicht inkludiert wurden. Offenbar war also das spezifisch stimmbezogene Leiden bei nicht von einer COVID-Erkrankung betroffenen Patienten unabhängig vom Pandemiegeschehen. In der aktuellen Literatur finden sich bisher dazu keine Berichte.

Das selbst empfundene Ausmaß der Stimmstörung unterlag im Gesamtkollektiv und in den einzelnen Quartalen keiner Geschlechtsspezifität. Im Gesamtkollektiv sowie von Männern wurde das Ausmaß der Dysphonie mit zunehmendem Alter höher eingeschätzt, nicht aber von Frauen. Die Ursache dafür ist spekulativ.

Nicht in den einzelnen Quartalen, aber im Gesamtkollektiv waren im Fragebogen zur stimmbezogenen Lebensqualität die Prozentwerte von Frauen mit 68,6 ± 22,5 % statistisch signifikant niedriger als bei Männern mit 73,4 ± 21,5 %. In der Literatur stellt sich dieses Antwortverhalten im V‑RQoL eher nicht als geschlechtsspezifisch dar [25, 27, 30], die klinische Bedeutung des aktuellen Befundes wird angesichts der sich deutlich überlappenden Standardabweichungen für die Betrachtung eines Einzelfalls als wenig bedeutsam eingeschätzt.

Das individuelle Ausmaß in den Domänen Depressivität, Ängstlichkeit und Stress war in dieser Studie während unverändert gegenüber vor und nach der Pandemie. Dabei wurden auch keine altersabhängigen Unterschiede gesehen, anders als bei [14, 29]: Mögliche konfundierende Faktoren wie die Lebensform, die Wohnungssituation u.a.m. wurden in dieser Studie nicht systematisch erfasst, sie könnten die Unterschiede zu den Ergebnissen anderer Arbeitsgruppen begründen. In der Domäne Ängstlichkeit hatten Frauen auffälligere Werte als Männer, nicht aber in der Depressivität; grundsätzlich haben Frauen ein zwei- bis dreifach höheres Risiko der Entwicklung einer spezifischen emotionalen Störung. Das an dieser Stelle nicht erwartete Ergebnis mag darin begründet sein, dass das Patientenkollektiv dieser Studie die tatsächlich in einer spezialisierten Sprechstunde erschienenen Patienten abbildet und keinen Bevölkerungsquerschnitt, der davon abweichen kann.

Die emotionalen Domänen Depressivität und Ängstlichkeit variierten im Beobachtungszeitraum nicht signifikant, aber die Domäne Stress, jedoch ohne erkennbaren Bezug zum Pandemieverlauf. Daraus kann keine störungs- und pandemiespezifische Folgerung hergleitet werden, sondern lediglich der individualdiagnostische Wert des Verfahrens bestätigt werden.

Die Ergebnisse beider eingesetzten Fragebögen korrelierten statistisch signifikant. Ein Ursachengefüge lässt sich daraus nicht herleiten. Indes belegt der Zusammenhang die Sinnhaftigkeit eines umfänglichen diagnostischen Zugangs bei Patienten mit Dysphonien. Das gilt nicht nur für Patienten mit funktionellen Dysphonien, sondern nach den Ergebnissen früherer Studien [11] auch für solche mit organischen Erkrankungen. Die eingesetzten Fragebögen können in ihrer Gesamtheit somit auch als Indikationsparameter für Maßnahmen der psychosomatischen Grundversorgung dienen. Der Zusammenhang zwischen dem stimmbezogenen Leiden und anderen psychischen Aspekten bestätigt Angaben in der Literatur, z. B. [14, 29].

Der mögliche Einfluss einer beruflichen Stimmbelastung (in Abhängigkeit von Tages- und Wochenarbeitszeit, Gebrauch von Schutzmasken, Raumakustik und -klima) wurde in dieser retrospektiven Studie nicht spezifisch erhoben und bewertet.

Weiterer Forschungsbedarf besteht auch jenseits von der Pandemie zur stimmbezogenen Lebensqualität und zu emotionalen Domänen bei Kindern und Jugendlichen, bei betagten und/oder in spezialisierten Einrichtungen lebenden Senioren und auch bei nicht stimmlich besonders beanspruchten Berufsgruppen. Dazu wären auch wegen des Erreichens einer adäquaten Fallzahl von Subgruppen weitere, ggf. auch multizentrische Untersuchungen erforderlich.

## Fazit für die Praxis

- Die coronabedingten Lockdowns beeinflussten die Patientenzahlen bei funktionellen Dysphonien nur zu Beginn der Pandemie.
- Das Inanspruchnahmeverhalten war weder alters- noch geschlechtsabhängig.
- Das Leiden der Patienten, operationalisiert durch die „stimmbezogene Lebensqualität“ und die Domänen „Depressivität, Ängstlichkeit und Stress“, war während der Pandemie unverändert gegenüber den Zeiträumen davor und danach.
- Die stimmbezogene Lebensqualität und die emotionalen Domänen korrelierten statistisch signifikant.

## Data Availability

Die Daten der Studie werden auf Anfrage verfügbar gemacht.
